# Latent autoimmune diabetes of adulthood: case report

**DOI:** 10.1186/s40842-017-0049-9

**Published:** 2017-11-28

**Authors:** Cristen P. Page, Brian Fitzgerald, Emily M. Hawes

**Affiliations:** 1 University of North Carolina (UNC) Family Medicine, 590 Manning Drive, CB#7595, Chapel Hill, NC 27514 USA; 2 Moncure Community Health Center-Piedmont Health Services, 7228 Moncure-Pittsboro Road, P.O. Box 319, Moncure, NC 27559 USA; 3 UNC Eshelman School of Pharmacy, 301 Pharmacy Ln, Chapel Hill, NC 27599 USA

**Keywords:** Diabetes, Graves’ disease, Autoimmune, Endocrine, Polyglandular autoimmune syndromes, Polydipsia, Diabetic ketoacidosis

## Abstract

**Background:**

Primary care clinicians will see a higher incidence of type 2 diabetes in adult patients, and the diagnosis and management of an initial presentation of type 1 diabetes can pose challenges to clinicians who see it less frequently. Symptoms of hyperglycemia and risk of ketoacidosis may be missed. Further, endocrine autoimmune disease can run together in patients and families.

**Case presentation:**

A 49-year-old Caucasian female with history of pituitary adenoma and Graves’ disease with history of thyroid ablation presented in the outpatient setting due to hand tingling of her right middle finger that was worse in the mornings and improved throughout the day. She also complained of excessive thirst, finding herself drinking more water than usual and waking up in the night to urinate. There was no dysuria or haematuria, and no other neurologic symptoms. She did report feeling hungry. She had no family history of diabetes, normal body mass index of 21.7, and reported taking her thyroid replacement medication every day. The differential diagnosis for her thirst included dehydration, psychogenic polydipsia, diabetes mellitus, diabetes insipidus, and anxiety. The patient had normal vital signs and was well appearing; labs were ordered for her on her way home from clinic with no medications. Labs revealed a random blood glucose level of 249 mg/dL, normal renal function, a normal B12 of 996 pg/mL, and an elevated thyroid stimulating hormone level of 25.67 u[iU]/mL. On follow up with her primary care provider 5 days later, additional labs were drawn showing A1C of 11.5%, 1+ ketonuria, a negative Acetest, and a normal basic metabolic panel, except for a fasting glucose of 248 mg/dL, and Free T3 of 2.42 pg/mL, and Free T4 of 1.7 ng/dL. Islet cell antibodies and glutamic acid decarboxylase antibodies were both positive, consistent with type 1 diabetes. She was started on insulin and improved.

**Conclusion:**

Given the patient’s age, this is a less common presentation of type 1 diabetes mellitus, as a part of polyglandular autoimmune syndrome type IIIa. It serves as a reminder that clinicians should remember that patients with one autoimmune disease (in this case, h/o Graves’ disease) are at higher risk for diabetes and other endocrine autoimmune diseases and should be screened appropriately. Clinicians should keep latent type 1 diabetes in the differential in adulthood to ensure proper and timely treatment.

## Background

While diabetes is an extremely common disease, most primary care clinicians will see more type 2 diabetes in adult patients, and the diagnosis and management of an initial presentation of type 1 diabetes can pose challenges to clinicians who see it less frequently. This case offers a reminder that a random blood glucose over 200 and classic hyperglycemic symptoms of polyuria, polydypsia, and nocturia were sufficient for the diagnosis of diabetes at the patient’s initial visit, whereas in the absence of unequivocal hyperglycemia, a test diagnostic of diabetes should be confirmed by repeat testing [1]. Additionally, this patient was at higher risk for type 1 diabetes given her history of Graves’ disease, an important relationship for clinicians to recognize [2]. While the majority of patients in her age group present with type 2 diabetes, she presented with classic symptoms of type 1 diabetes [3].

Type 1 diabetes is autoimmune in 95% of cases (type 1a) and idiopathic in under 5% of cases (type 1b). While type 1 diabetes can present at any age, peaks in incidence occur before school age and again around puberty [4]. Given that autoimmune destruction of beta cells has multiple genetic predispositions, patients with other autoimmune disorders such as Hashimoto’s thyroiditis, Graves’ disease, Addison’s disease, celiac disease, autoimmune hepatitis, and myasthenia gravis, should be screened for type 1 diabetes [1, 5, 6].

A coexistence of at least two autoimmune-mediated endocrinopathies is classified as polyglandular autoimmune syndromes (PAS). Autoimmune thyroid disorders represent the most frequent autoimmune endocrinopathies within PAS and with a prevalence of 70 to 75%. The second most common endocrine autoimmune condition in the adult PAS patient is type 1 diabetes and is present in 50 to 60% of these patients [6].

Type 1 diabetes is defined by fasting glucose >126 mg/dL or postprandial glucose >200 mg/dL or hemoglobin A1c over 6.5% and insulin deficiency as well as clinical signs of an insulin deficiency syndrome (polyuria, polydipsia, weight loss, ketoacidosis). According to the American Diabetes Association, blood glucose rather than an A1c should be used to diagnose type 1 diabetes in patients with symptoms of hyperglycemia. Type 1 diabetes is defined by the presence of one or more diabetes-associated autoimmune markers, such as islet cell antibody (ICA) and glutamic acid decarboxylase antibody (GADA). If a new diagnosis of type 1 diabetes is suspected, diabetic ketoacidosis should be ruled out at the initial clinical presentation, given 25% of type 1 diabetes patients present in diabetic ketoacidosis [1, 5].

The degree of beta cell destruction in patients with type 1 diabetes is variable, although it is often considered more rapid in younger patients and slower in adults. Older adults can frequently present with a more indolent onset of type 1 diabetes, which may lead to misdiagnosis as type 2 diabetes. This particular case presentation is referenced by the term latent autoimmune diabetes of adulthood (LADA). LADA is defined by three criteria identified by the Immunology of Diabetes Society including adult age of onset (>30 years of age), presence of at least one circulating autoantibody (GADA/ICA/IAA/IA-2), and initial insulin independence (within the first 6 months) [7, 8].

Although LADA has similar genetic and immunologic features as juvenile-onset type 1 diabetes, it also embodies some characteristics of type 2 diabetes. At diagnosis, insulin secretion is often similar in patients with LADA and type 2 diabetes. These patients may have enough beta cell function that they may be controlled with oral medicines and diet initially, but over time develop increasing dependence on insulin therapy, and thus often require insulin earlier than would otherwise be expected in a type 2 diabetic. Compared to type 2 diabetes, patients with LADA require insulin treatment earlier and more commonly post diagnosis and show worse glycemic control than type 2 diabetes patients [7, 8].

Importantly, type 1 diabetes involves the progressive destruction of beta cells and therefore lower insulin levels, which predisposes these patients to developing ketoacidosis, often leading to pronounced hyperglycemia, and necessitates prompt treatment [3]. These patients have different treatment considerations than those with type 2 diabetes, and they more often present with complications like diabetic ketoacidosis [3]. This patient was not in ketoacidosis, but the lack of full evaluation on initial presentation delayed proper and timely treatment, which would have been a significant mistake had she been acidotic. It is important for clinicians to screen at risk patients as well as diagnose and manage type 1 diabetes efficiently even when it presents in an atypical fashion.

## Case presentation

This 49-year-old patient presented with a chief complaint of recent onset finger tingling, and also complained of excessive thirst, which was most pronounced at night. Although she normally did not drink much water, she was drinking about 1 gal of water per night. Additionally, she reported frequent urination, having to wake up six to eight times per night to urinate. She also reported being hungry after dinner, which was abnormal for her. She denied nausea or vomiting. The patient had no known family history of diabetes, but did have personal history of pituitary adenoma (diagnosed at age 18 without progression) and history of Graves’ disease with history of thyroid ablation 10 years previously. Her only other past history included biceps tendonitis and left shoulder pain. Her medications included levothyroxine 137mcg 4 days per week and 125mcg 3 days per week. She reported that on the days when she took 137 mcg of levothyroxine, she noticed her heart beating faster. She was very physically active as part of her job as a fitness instructor, and she reported feeling as though she might pass out when she was kneeling down and then standing up.

Objectively, patient’s BMI at presentation was 21.7 kg/m^2^. She lost 1.27 kg in the 10 days prior to presentation to clinic. Her heart rate was 96 beats per minute. Otherwise, her physical exam was unremarkable.

This patient had multiple features suggesting that she should be tested for autoantibodies, which would help to confirm the diagnosis of type 1 diabetes. These features included age of onset <50 years, acute symptoms, BMI <25, and personal or family history of autoimmune disease [4]. Some clinicians measure two antibodies (glutamic acid decarboxylase and islet cell antibodies), and especially if both antibodies are positive, the patient should be presumed to have type 1 diabetes and often initiated on insulin [4, 5, 9].

About 25% of adults newly diagnosed with type 1 diabetes can be in diabetic ketoacidosis [3]. Thus, this should be ruled out at initial presentation in patients presenting with an insulin deficiency syndrome. While this ideally would have been done at the patient’s initial visit when she was found to be hyperglycemic, further testing was not performed at that time. At the patient’s follow up visit five days later, a basic metabolic panel was done, which ruled out an anion gap metabolic acidosis, and a urinalysis was performed, which confirmed glucosuria and ketonuria. An Acetest (nitroprusside reaction to measure levels of serum acetone and acetate) was done to rule out serum ketone production, and this test was negative. This test is limited in evaluation for serum ketones because it does not test for beta-hydroxybuturate, another serum ketone produced in DKA. However, at our institution, testing for beta-hydroxybutyrate does not result for 2–3 days, thus it would have been unlikely to change our initial management.

The patient’s ICA was 0.06 nmol/L (reference range < =0.02 nmol/L) and GADA was 2.81 (reference range < =0.02 nmol/L). Of note, GADA is often used interchangeably with glutamic acid decarboxylase 65 (GAD 65). In newly diagnosed patients with type 1 diabetes, GADA has a sensitivity of 70–90% and specificity 99% for the diagnosis, and islet cell antibody testing has a sensitivity of 44–100% and specificity of 96% [10]. Also, GAD 65 has a positive predictive value of 92% for requiring insulin at three years in persons 15 to 34 years of age, and ICA has a positive predictive value of 86% for requiring insulin at three years in persons 15 to 34 years of age [11]. Thus, each of these tests confers an increased likelihood of requiring insulin. In addition, the magnitude of the insulin response is inversely proportional to the GAD levels [7].

Given the near total deficiency of insulin and subsequent risk of ketoacidosis, insulin is often initiated at diagnosis and is considered the mainstay of therapy for patients with type 1 diabetes. For patients with type 1 diabetes, the goal of insulin therapy is to provide a physiologic profile of insulin by giving a daily (or twice daily) long acting insulin preparation as well as pre-meal boluses of a rapid acting insulin. Our patient was started on long acting insulin glargine 10 units at night and rapid acting insulin lispro 2 units with breakfast, lunch, and dinner. Initial total insulin dose in type 1 diabetic patients is 0.2 to 0.6 units/kg/day, but in order to avoid complications of hypoglycemia, it is reasonable to start with 0.2–0.4 units/kg/day. Thus, our patient was started on approximately 0.3 units/kg/day of insulin in total [5, 9, 12].

With an insulin regimen of insulin glargine and lispro before meals, her Hemoglobin A1c came down to 7.6% over the next 9 months. She was started on a statin and aspirin for cardiovascular risk reduction. At one year after presentation, her treatment team included her primary care physician, endocrinologist, nutritionist, and pharmacist. Her medication regimen was insulin glargine 10 units/sc at night, insulin lispro 3 units before meals, Atorvastatin 10 mg daily, and aspirin 81 mg daily. Self-monitoring glucose was a 7-day average of 139 mg/dL and a 14-day of average 144 mg/dL.

## Discussion and conclusions

Diabetes was at the top of the differential diagnosis list upon presentation. This patient was likely to have type 1 diabetes at presentation, as defined by fasting glucose >126 or postprandial glucose >200, or hemoglobin A1c of 6.5% or greater and insulin deficiency, as well as clinical signs of an insulin deficiency syndrome (polyuria, polydipsia, weight loss, ketoacidosis [8]. We had discovered all of these features by the time of her follow up visit, except testing for C-peptide was not done for insulin deficiency. The patient’s polyphagia with weight loss and nocturnal enuresis are all clinical features of type 1 diabetes at diagnosis and not type 2 diabetes. The patient’s report of presyncopal symptoms also fit with volume depletion caused by osmotic diuresis due to hyperglycemia. In contrast, patients with type 2 diabetes are often asymptomatic at presentation [13].

Another diagnostic consideration was overtreatment of her hypothyroidism. Her weight loss, increased appetite, urinary frequency, and more rapid heartbeat could have been explained by hyperthyroidism [14]. However, her elevated TSH was consistent with under treatment and subsequent free T-4 was normal.

In a patient with multiple autoimmune diseases, the clinician should have a higher index of suspicion that the patient could present with an additional autoimmune disease. Our patient could be classified as having one of the polyglandular autoimmune syndromes (PAS), specifically PAS IIIa [6, 15]. These syndromes are divided into PAS I, II, and III. While juvenile PAS I is quite rare, PAS II commonly presents in the third or fourth decade in women, and has a prevalence of 1:20,000. The main difference between PAS III and PAS II is the absence of adrenal cortical involvement causing adrenal insufficiency in PAS III, while this is present in PAS II. Autoimmune thyroiditis (Graves’ disease or Hashimoto’s thyroiditis) and type 1 diabetes, when occurring together, are defined as autoimmune polyglandular syndrome type IIIa [15]. Notably, there is often a long time interval between the manifestation of the first and second component disease of PAS II, which often comprises years to decades. Perhaps our patient may later develop adrenal insufficiency given she has both type 1 diabetes and autoimmune thyroid disease, which may suggest an underlying genetic predisposition to PAS II. PAS II can also include features of adrenal insufficiency, hypoparathyroidism, primary hypogonadism, and other manifestations [6]. Clinicians need to be on the lookout for these associated diseases [6, 16, 17].

Experts recommend serologic and functional screening for these associated diseases in patients with a monoglandular autoimmune disease at diagnosis and during follow-up appointments at least every 2 years [6, 16]. Although diabetes-associated autoantibodies can be obtained to predict individuals at risk for developing type 1 diabetes, methods to delay or prevent disease onset are not promising. Immunosuppressive agents (such as cyclosporine, rituximab, abatacept), which come with a variety side effects, have been evaluated to prevent or delay type 1 diabetes and shown limited to no impact. For the few drugs that appear to have some effect, not all patients respond and for those who do, the effects are often short-lived. Although there is limited evaluation of beta-cell prevention or preservation in LADA patients, one study using a GAD-alum formulation demonstrated a relative preservation of C-peptide release for 5 years, with limited side effects. Still, the benefit of screening diabetes-associated autoantibodies prior to diagnosis remains controversial [7, 18]. Given that our patient has type 1 diabetes and thyroid disease, screening may include functional assessment of adrenal failure, primary hypogonadism, hypoparathyroidism, type A autoimmune gastritis with or without pernicious anemia, and celiac disease.

After identification of autoimmune thyroid disease, screening for diabetes (such as oral glucose tolerance testing) should have been implemented and could have led to earlier detection in our patient. Our patient was started on insulin at diagnosis and given that her presentation was more consistent with type 1 diabetes versus early onset LADA. Insulin is often started in patients who have weight loss or dehydration in the setting of hyperglycemia or if they have evidence of increased ketogenesis as evidenced by ketonuria or acidosis; our patient met all of these criteria aside from the acidosis and thus we started her on insulin [4, 9]. Interestingly, our patient may have had LADA for years before this clinical presentation, causing her beta cell mass to decrease, which resulted in having more features of type 1 diabetes than other adult patients with LADA, who do not require insulin at diagnosis [4, 7]. Previously, a blood glucose had not been checked to screen for diabetes. If her clinicians had been more mindful of the relationship between autoimmune thyroid disease and diabetes, perhaps they would have checked her blood sugar sooner (despite her appearance as a fit, healthy female), diagnosed LADA, and potentially delayed her progression to insulin dependence through early treatment.

Due to the paucity of data, the optimal initial treatment of latent autoimmune diabetes in adults (LADA) is not established. Studies treating recently-diagnosed LADA patients with dipeptidyl peptidase 4 (DPP-4) inhibitors suggest that these may postpone loss of beta cell function, although these findings are not convincing and need to be confirmed in larger randomized trials. Despite its widespread use in type 2 disease, there are no studies evaluating the impact of metformin alone in patients with LADA. However, use of sulfonylureas is discouraged due to rapid deterioration of beta cells [7]. To date, the evidence suggests that patients with LADA should be initially treated with insulin when glycemic control declines to a level indicating need for antidiabetic treatment [7, 19].

Unfortunately, treatment of T1DM remains an ongoing challenge. Tight control with intensive insulin treatment leading to reduced complications is often hindered by increased hypoglycemia and weight gain. Physiologic insulin strategies with rapid-acting analog insulins and insulin pumps combined with continuous glucose monitoring systems have shown improved glycemic control and decreased side effects. Use of adjunctive therapies, with non-insulin therapies, can be considered for patients with type 1 diabetes failing to achieve glycemic control on insulin [5, 9, 20].

Pramlintide, an amylin analog, is the only FDA-approved adjunctive therapy for type 1 diabetes. Use is limited due to concerns with tolerability and the need for administration by multiple daily injections or an infusion pump [5, 20]. Evidence suggests that adding metformin to insulin therapy may reduce insulin doses and improve metabolic control in overweight type 1 diabetic patients [20, 21]. Promising data is available for glucagon-like peptide 1 (GLP-1) receptor agonists and dipeptidyl peptidase 4 (DPP-4) inhibitors due to their potential protection of beta cells and suppression of glucagon release [20]. More clinical data exist for GLP-1 agonists which show a substantial reduction in post-prandial glucose excursions, weight and daily insulin requirements [20, 22, 23]. Sodium–glucose cotransporter 2 (SGLT2) inhibitors provide insulin-independent glucose lowering by inhibiting glucose reabsorption in the proximal renal tubule. The FDA has warned about the risk of ketoacidosis occurring in the absence of significant hyperglycemia in patients receiving these medications; thus, patients should be counseled on maintaining adequate hydration and monitored for ketoacidosis [20, 24]. Furthermore, patients should be warned that the FDA recently issued a boxed warning for increased risk for leg and foot amputation for one SGLT2 inhibitor, Canagliflozin. A clear benefit has not been demonstrated with use of thiazolidinediones (TZDs) in type 1 patients and the many potential side effects make it a less appealing option for add-on therapy. Use of metformin, GLP-1 agonists, DPP-4 inhibitors, and SGLT2 inhibitors may be considered safe and effective add-on therapy to insulin although large clinical trials are needed before routine use in this population [20]. Our patient may have benefited from one of these therapies, which highlights that primary care providers need a broader understanding of acceptable treatment options for patients with LADA.

A previous autoimmune disease in a given patient increases the chance of another autoimmune disease in that same patient, and therefore there should be a low threshold for screening for concomitant autoimmune diseases. Screening and regular follow-up should be performed to detect other endocrine deficiencies before the development of potentially severe acute (such as adrenal crisis or ketoacidosis) or chronic complications (such as microvascular damage in type 1 diabetes). Early awareness of patients having LADA may result in more regular and targeted follow-up and a quicker transition to insulin if not initiated at diagnosis. The mainstay of therapy is insulin replacement, although tight control with intensive insulin is challenging. Insulin resistance is increasingly recognized as a barrier to ideal glycemic control in LADA, thus non-insulin therapies can be considered at early diagnosis or more commonly as add-on to insulin.

## Abbreviations

BMI: Body mass index
DKA: Diabetic ketoacidosis
GAD 65: Glutamic acid decarboxylase 65
GADA: Glutamic acid decarboxylase antibody
IAA: Insulin autoantibodies
ICA: Islet cell antibody
LADA: Term latent autoimmune diabetes of adulthood
PAS: Polyglandular autoimmune syndromes
TSH: Thyroid stimulating hormone

## References

[1] American Diabetes Association. Classification and diagnosis of diabetes. Sec. 2. In Standards of Medical Care in Diabetes 2017. Diabetes Care 2017;40(Suppl. 1):S11–S24. 10.2337/dc17-S005 27979889

[2] White RD, Harris GD (2006). Case study: “birds of a feather flock together”: type 1A diabetes and other autoimmune disease states. Clin Diabetes.

[3] McCullough DK. Clinical presentation and diagnosis of diabetes mellitus. In: UpToDate, Post TW (Ed), UpToDate, Waltham, MA. Accessed 20 September 2017.

[4] McCullough DK. Classification of diabetes mellitus and genetic diabetic syndromes. In: UpToDate, Post TW (Ed), UpToDate, Waltham, MA. Accessed 20 September 2017.

[5] Chiang JL, Kirkman MS, Laffel LM, Peters AL (2014). Type 1 Diabetes Sourcebook Authors. Type 1 diabetes through the life span: a position statement of the American Diabetes Association. Diabetes Care.

[6] Kahaly GJ, Frommer L. Polyglandular autoimmune syndromes. J Endocrinol Investig 2017 . doi: 10.1007/s40618-017-0740-9. [Epub ahead of print] Review. 10.1007/s40618-017-0740-9 28819917

[7] Laugesen E, Østergaard JA, Leslie RD (2015). Danish Diabetes Academy Workshop and Workshop Speakers. Latent autoimmune diabetes of the adult: current knowledge and uncertainty. Diabet Med.

[8] Merger SR, Leslie RD, Boehm BO (2013). The broad clinical phenotype of type 1 diabetes at presentation. Diabet Med.

[9] American Diabetes Association (2017). Pharmacologic approaches to glycemic treatment. Sec. 8. In standards of medical Care in Diabetes 2017. Diabetes Care.

[10] Masharani U, Papadakis MA, McPhee SJ, Rabow MW (2014). Diabetes mellitus & hypoglycemia. Current Medical Diagnosis & Treatment 2015.

[11] Törn C, Landin-Olsson M, Ostman J (2000). Glutamic acid decarboxylase antibodies (GADA) is the most important factor for prediction of insulin therapy within 3 years in young adult diabetic patients not classified as type 1 diabetes on clinical grounds. Diabetes Metab Res Rev.

[12] Lexicomp Online ®, Insulin lispro: Drug information. Insulin Lispro, Lexi-Drugs®, Hudson, Ohio: Lexi-Comp, Inc.;2015. https://online.lexi.com/lco/action/doc/retrieve/docid/fc_dfc/5548571. Accessed 20 Sept 2017.

[13] Masharani U, German MS. Pancreatic hormones and diabetes mellitus. In: Gardner DG, Shoback D. editors. Greenspan’s Basic & Clinical Endocrinology, 9e*.* New York: McGraw-Hill; 2011. http://accessmedicine.mhmedical.com/content.aspx?bookid=380§ionid=39744057. Accessed 20 Sept 2017.

[14] Ross DS. Diagnosis of hyperthyroidism. In: UpToDate, Post TW (Ed), UpToDate, Waltham, MA. Accessed 20 Sept 2017.

[15] Thakkar UG, Vanikar AV, Trivedi HL (2013). Polyglandular autoimmune syndrome type III: two cases. Sri Lanka J Child Health.

[16] Hansen MP, Matheis N, Kahaly GJ (2015). Type 1 diabetes and polyglandular autoimmune syndrome: a review. World J Diabetes.

[17] Dittmar M, Kahaly GJ (2010). Genetics of the autoimmune polyglandular syndrome type 3 variant. Thyroid.

[18] Simmons KM, Gottlieb PA, Michels AW (2016). Immune intervention and preservation of pancreatic Beta cell function in type 1 diabetes. Curr Diab Rep..

[19] Thunander M, Thorgeirsson H, Törn C, Petersson C, Landin-Olsson M (2011). β-cell function and metabolic control in latent autoimmune diabetes in adults with early insulin versus conventional treatment: a 3-year follow-up. Eur J Endocrinol.

[20] Munir KM, Davis SN (2015). The treatment of type 1 diabetes mellitus with agents approved for type 2 diabetes mellitus. Expert Opin Pharmacother.

[21] Livingstone R, Boyle JG, Petrie JR, REMOVAL Study Team. A new perspective on metformin therapy in type 1 diabetes. Diabetologia. 2017; 10.1007/s00125-017-4364-6. 10.1007/s00125-017-4364-6 PMC5552844 28770327

[22] Ghazi T (2014). Rink, Sherr JL, Herold KC. Acute metabolic effects of exenatide in patients with type 1 diabetes with and without residual insulin to oral and intravenous glucose challenges. Diabetes Care.

[23] Mottalib A, Kasetty M, Mar JY (2017). Weight Management in Patients with type 1 diabetes and obesity. Curr Diab Rep.

[24] Perkins BA, Cherney DZ, Partridge H (2014). Sodium-glucose co transporter 2 inhibition and glycemic control in type 1 diabetes: results of an 8-week open label proof-of-concept trial. DiabetesCare.

